# Primary Sweat Gland Adenocarcinoma of the Skin With *ATL2::PRKD3* Fusion: A Potential Cutaneous Analog of Cribriform Adenocarcinoma of the Salivary Glands?

**DOI:** 10.1002/gcc.70093

**Published:** 2025-11-12

**Authors:** Anaïs Brunet, Barouyr Baroudjian, Maxence Mancini, Fanélie Jouenne, Baptiste Louveau, Isabelle Moulonguet, Amélie Osio, Samia Mourah, Maxime Battistella

**Affiliations:** ^1^ Pathology Department AP‐HP Hôpital Saint‐Louis Paris France; ^2^ Department of Dermatology AP‐HP Hôpital Saint‐Louis Paris France; ^3^ Tumor Genomics and Pharmacology Department AP‐HP Hôpital Saint‐Louis Paris France; ^4^ Université Paris Cité, INSERM U1342 Paris France

**Keywords:** adnexal adenocarcinoma, cribriform adenocarcinoma of salivary gland, cribriform subtype of polymorphous adenocarcinoma, PRKD3, sweat gland adenocarcinoma

## Abstract

Many cutaneous adnexal tumors share molecular alterations with other homologous neoplasms occurring in salivary glands. Polymorphous adenocarcinoma (PAC) is a rare salivary gland tumor, usually associated with alterations in the *PRKD* gene family. In this report, we describe a primary cutaneous sweat gland adenocarcinoma of the scalp occurring in a 65‐year‐old female. Morphological features of the carcinoma were reminiscent of salivary gland PAC. Tumor cells were CK7+ S100+ SOX10+ p63 focally positive and p40‐. Whole‐transcriptome sequencing of the lesion showed the presence of an in‐frame *ATL2::PRKD3* fusion. *PRKD1*, *2* or *3* fusions are characteristic of the cribriform subtype of PAC (cribriform adenocarcinoma of salivary gland, CASG). No past history of salivary gland tumor nor existing salivary gland tumor was retrieved in the patient. After complete excision of the tumor, the patient has been in complete remission for 17 months. This case suggests that a subset of adnexal adenocarcinomas of not otherwise specified type (NOS) may carry *PRKD* alteration and may constitute the cutaneous counterpart of salivary gland PAC/CASG. This finding argues in favor of a more systematic molecular exploration of adnexal adenocarcinomas NOS for better classification and prognosis.

## 1 Introduction

Cutaneous sweat gland tumors form a heterogeneous group of neoplasms whose diversity makes the diagnosis difficult for pathologists. Recent advances in genomic analysis have enabled better characterization and classification of adnexal tumors, and new molecular‐defined subtypes are recurrently identified [1]. Several skin adnexal tumor entities are the cutaneous homologs of salivary tumors, that share similar morphology and molecular alterations [2]. The discovery of genomic alterations in salivary gland tumors has allowed the characterization of several entities [3, 4]. Cutaneous mixed tumor and pleomorphic adenoma have recurrent *PLAG1* fusions [5], and hidradenoma harbors *CRTC1::MAML2* fusion similar to mucoepidermoid carcinoma [2]. As in other organs, primary cutaneous adenoid cystic carcinomas carry *MYB* or *MYBL1* fusions, and primary cutaneous secretory carcinoma has *ETV6::NTRK3* fusions [2].

Polymorphous adenocarcinoma (PAC) is a common, usually low‐grade salivary gland carcinoma, with an overlapping spectrum of morphology with cribriform adenocarcinoma of salivary gland (CASG), which is considered by some authors as a subtype of PAC [6]. These tumors are more common in women around their sixth decade and both share genetic alterations of PRKD genes. PAC is characterized by a recurrent *PRKD1* E710D mutation while CASG harbors rearrangements involving *PRKD1*, *PRKD2* or *PRKD3* genes. Beyond these molecular alterations, these 2 subtypes have slightly different histomorphology, clinical presentation, and prognosis. Contrary to PAC which has an indolent behavior, CASG displays a higher risk of lymph node metastasis. However, this distinction is not absolute [7, 8, 9] and there is still controversy on whether CASG is a subtype of PAC or a different entity.

Here, we report a case of a 65‐year‐old woman presenting with a primary skin adenocarcinoma of the scalp harboring an *ATL2::PRKD3* fusion transcript. This rearrangement has already been described in CASG, but to our knowledge this is the first reported case of a primary skin tumor harboring this molecular alteration, which suggests the existence of a cutaneous counterpart of salivary PAC/CASG.

## 2 Materials and Methods

### 2.1 Immunohistochemistry

Immunohistochemistry was performed on a Benchmark Ultra (Roche) autostainer according to the standard protocols, using the following primary antibodies: CD117 (polyclonal, Agilent, 1:100), CK7 (clone OV‐TL 12/30, Agilent, 1:100), estrogen receptor (clone SP1, Roche, RTU), GATA3 (clone L50‐823, Diagomics BioSB, pre‐diluted), HER2 (clone 4B5, Roche, RTU), p63 (clone 4A4, Roche, RTU), p40 (polyclonal, Clinisciences, 1:50), S100 (polyclonal, Agilent, RTU), SOX10 (clone SP267, Roche, RTU).

### 2.2 RNAseq Whole Transcriptome Sequencing

RNA was extracted from FFPE tissue sections by using a Maxwell 16 Instrument (Promega, Madison, WI, USA) with the Maxwell RSC FFPE RNA kit. Library production was conducted with SureSelect XTHS2 technology (Agilent, Santa Clara, CA, USA). Qualification of purified libraries was assessed with Bioanalyzer (DNA 1000 kit for 2100 Bioanalyzer Systems, Agilent). Molecular analysis was performed using a 101‐cycle paired‐end whole RNA sequencing on a Nextseq 2000 platform (Illumina). Sequencing results were obtained after fastq alignment to the GRCh38 human reference sequence and fusion calling was achieved with star_arriba, star_fusion and fusioncatcher, using G‐Route APHP RNA_SEQ 2.4.4 pipeline (MOABI—AP‐HP Bioinformatics Platform—WIND—DSI, Paris, France).

## 3 Case Presentation

### 3.1 Clinical and Histopathological Findings

A 65‐year‐old woman presented with a 1 cm nodular tumor of the scalp with an indeterminate duration. She had a past medical history of invasive breast carcinoma, not otherwise specified 20 years ago of the right breast treated by mastectomy and axillary lymph node dissection without recurrence.

**FIGURE 1:**
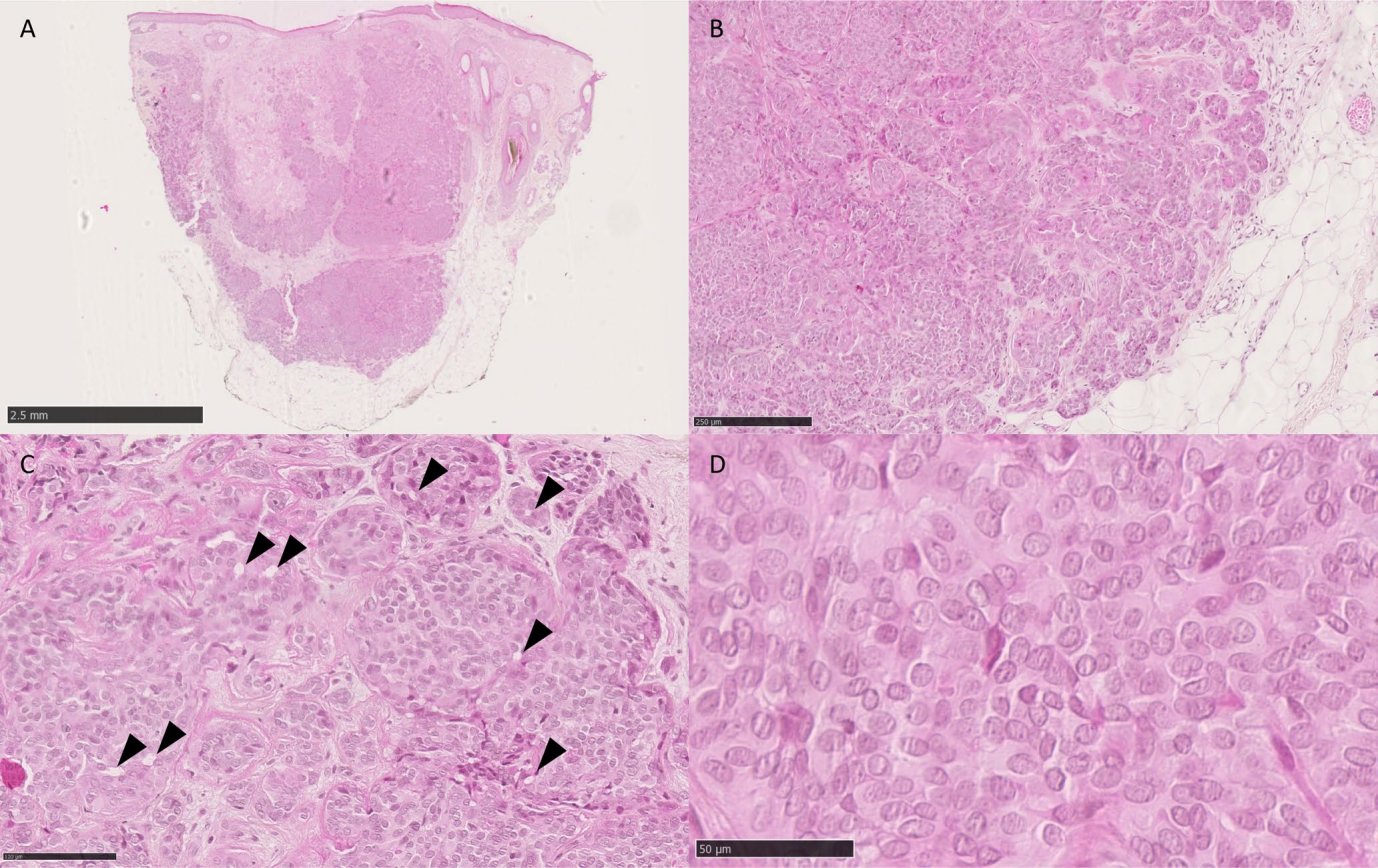
Morphological features. Biopsy showed a multinodular dermal and superficial subcutaneous tumor (A) without connection with the epidermis (B). Architecture was predominantly solid, sometimes with tubulo‐papillary areas at the periphery and infiltrative aspects (B). Some areas of cribriform architecture were observed (arrow heads) (C). Cells were monotonous with round‐oval bland nuclei and some clear nuclei (D).

**FIGURE 2:**
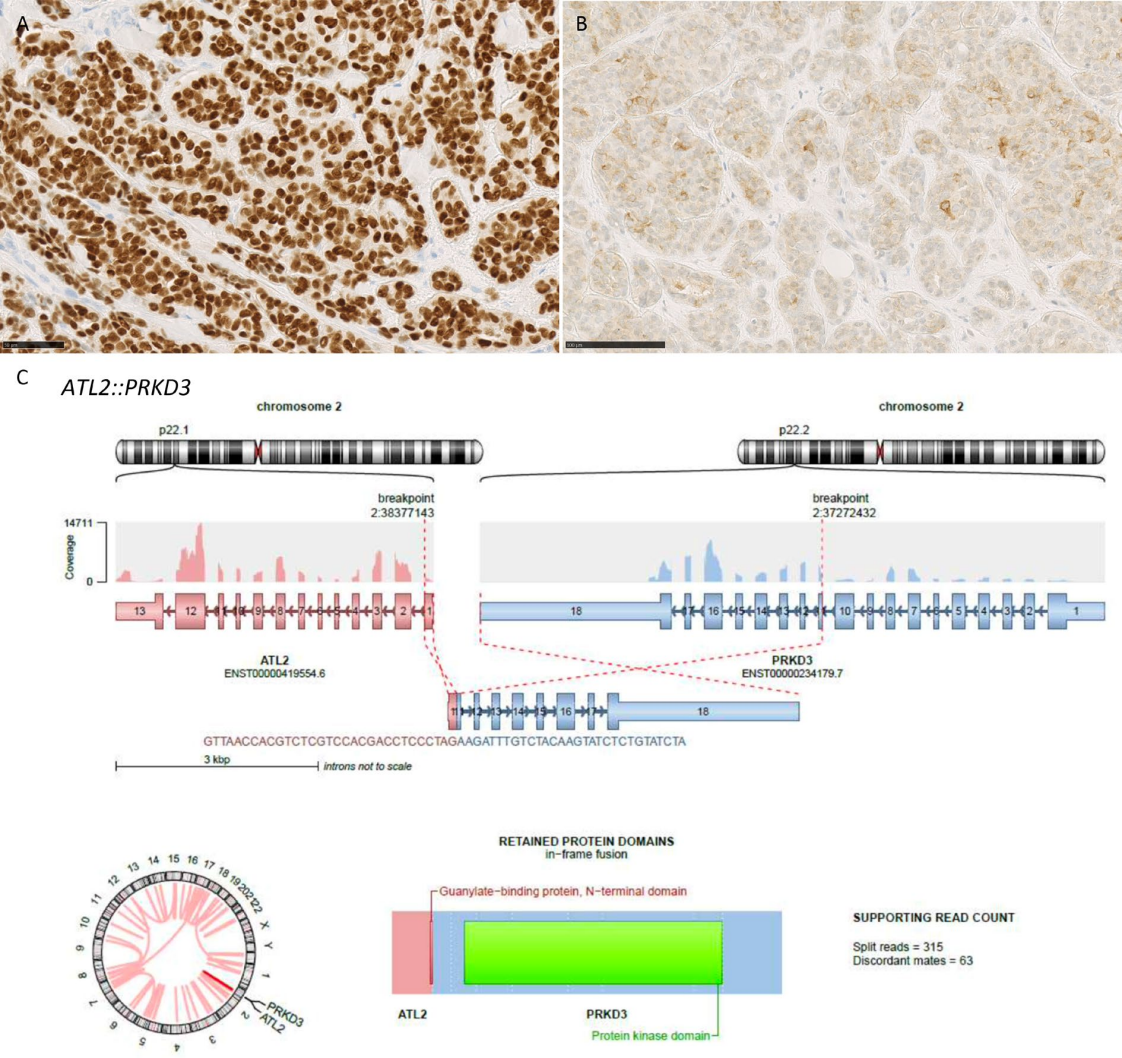
Immunohistochemical and molecular features. Tumor cells expressed diffusely SOX10 (A) with heterogeneous expression of CD117 (B); RNAseq whole transcriptome sequencing revealed an in‐frame *ATL2::PRKD3* fusion resulting in a fusion between the N‐terminal domain of *ATL2* and the PH domain of *PRKD3* (C). (generated with ARRIBA pipeline).

The nodule underwent surgical biopsy. Histologically, a dermal and superficial subcutaneous multinodular epithelial tumor without connection to the epidermis was seen (Figure 1A). It had a solid growth pattern, with some tubulo‐papillary areas (Figure 1B). Rare areas of cribriform architecture were observed (Figure 1C). The tumor was rather poorly limited, with infiltrative borders and large nodules separated by fibrous septa. Cytologically, cells were monomorphous, uniform in shape, with scant cytoplasm, bland round to oval nuclei, occasional small nucleoli (Figure 1D) with rare mitoses. Some clear nuclei were noted. There was no high‐grade area, no glomerular or papillary architecture. There was no necrosis or perineural or vascular invasion. Immunohistochemistry revealed a diffuse expression of CK7, SOX10 and S100 (Figure 2A), with focal expression of CD117 (Figure 2B). Only rare p63+ nuclei were noted, while p40 was negative. No GATA3 expression was present. Ten to twenty percent of cells were estrogen receptor‐positive with a 2+ intensity. Weak expression of HER2 was noted (1+).

The morphology and phenotype of this cutaneous tumor were different from that of the breast carcinoma, which was strongly and diffusely estrogen receptor‐positive and had a HER2 3+ expression without SOX10 expression.

Overall, based on morphology and immunohistochemical results, a working diagnosis of adnexal adenocarcinoma NOS was proposed and further molecular exploration was performed on the skin tumor's biopsy containing 95% of tumor cells.

Whole transcriptome sequencing revealed an in‐frame *ATL2::PRKD3* fusion (Figure 2C), which has been previously described in CASG [10]. High‐throughput DNA sequencing using a panel of 538 genes retrieved an intermediate tumor mutational burden of 13 mutations per megabase (mut/Mb) and identified 2 variants of unknown significance (VUS): *NRAS* (NM_002524) c.272C>T, (p.Ala91Val) (Variant allele frequency (VAF) of 45%); *ERBB3* (NM_001982) c.1739G>A, (p.Arg580Gln) (VAF 2%) as well as moderate copy number gains of *JAK2*, *KRAS* and *PIK3CA* (3 copies).

Genomic exploration of the previous breast cancer lesion (with 90% of tumor cells) showed a different molecular profile thus precluding a potential common tumor origin with the skin tumor: absence of the *PRKD* fusion, high tumor mutational burden (21 mut/Mb), truncating variation of *BCOR* (VAF 3%), amplifications of *ERBB2* (6 copies) and *CCND1* (4 copies), complete loss of *RB1* and several VUS. Only the *NRAS* alteration was also found in the primary breast tumor with a VAF of 40%, suggesting a germline origin.

The nodule of the scalp was then re‐excised with a 1 cm margin. Radiological workup by PET scan, cervical and breast echographies and mammography did not reveal any evidence of breast malignancy, nor salivary gland tumor. No recurrence was observed after 17 months.

Based on the morphological, immunohistochemical and molecular results a final diagnosis of sweat gland adenocarcinoma with *ATL2::PRKD3* fusion, a potential analog of cribriform adenocarcinoma of the salivary gland (CASG) was proposed.

## 4 Discussion

Here, we report the first case of primary cutaneous adnexal adenocarcinoma with *PRKD3* rearrangement, which suggests the existence of a cutaneous analog of CASG. Rearrangements of the *PRKD3* gene have been previously described in CASG, but only 3 studies have identified the rearrangement partner [10, 11, 12]. Interestingly, of the 4 CASG cases in which the rearrangement partner was identified, it was also *ATL2*, as in our case.

The *PRKD* gene family is involved in diacylglycerol and protein‐kinase C transduction pathways and shares high levels of sequence identity, particularly in the kinase domain. Specifically, the *PRKD* family members have an N‐terminal region with regulatory domains and a C‐terminal region with a kinase domain, the proteins being maintained in an inactive state through auto‐inhibition of the kinase domain by its regulatory domains. Rearrangements of the *PRKD3* gene, in our case and in the cases reported by Hahn et al. [11], are in‐frame and located between exons 10–13, containing the PH domain (Pleckstrin homology domain, involved in cell signaling and membrane‐associated processes). It results in a constitutive activation of the proteins encoded by the *PRKD* gene.

Atlastin 1 to 3 (ATL1‐3) are a family of membrane‐bound GTPase proteins located in the endoplasmic reticulum. ATL1 is expressed everywhere in the endoplasmic reticulum (ER), whereas ATL2 and ATL3 are mainly expressed at the ER junctions. Their main function is to join ER membrane tubules into a branched network. ATL proteins have an N‐terminal GTPase domain, necessary for membrane fusion and the generation of three‐way junctions, followed by a three‐helical bundle domain (3HB), two membrane domains, and a short α‐helical C‐terminus. ATL proteins also participate in the autophagic degradation of the ER membranes, regulate lipid droplet size and inhibit bone morphogenetic protein signaling [13, 14].

In androgen receptor positive breast cancer, increased ATL2 expression has been associated with worse prognosis [15]. In lung cancer, through a comparison of RNA networks in *EGFR* wild‐type and mutant non‐small cell lung cancer, it was correlated with shorter survival [16]. In our case, the breakpoint fusion is located in exon 1, containing the N‐terminal domain with the GTPase domain.

In the latest WHO classification of salivary gland [6], CASG are considered a subtype of PAC, with characteristic morphology and PRKD rearrangements. However, the distinction is not absolute. There are indeed tumors with intermediate or indeterminate morphology, illustrating the complexity of classifying PAC/CASG spectrum tumors and explaining the low correlation rate among pathologists in distinguishing between the different entities [8]. Similarly, several studies report that fusion or mutation was not exclusive to CASG or PAC 7–9 [7, 8, 9]. In our case, we observed only rare areas with a cribriform architecture.

Compared to PAC, CASG have a higher risk of metastasis. To date, our patient has not developed metastasis after 17 months, nor has shown any primary salivary gland tumor. It is possible that certain rearrangements of CASG are associated with a poorer prognosis. In Hahn's study, 4 cases out of 51 had high‐grade morphology and carried a *PRKD1* rearrangement [11]. Of the 3 cases with a *PRKD* family gene rearrangement, one case developed metastases and had a *PRKD1* rearrangement. Finally, there is a reported case of a CASG with high‐grade transformation carrying a *STRN3::PRKD1* fusion. Nevertheless, further data are needed to determine whether there is a correlation between the *PRKD* gene involved in the rearrangement and prognosis.

This case highlights the interest of molecular analysis on adnexal adenocarcinoma NOS, according to the latest WHO classification of skin tumors [17]. Indeed this basket category includes all cases of primary carcinoma of the skin with ductal/glandular differentiation lacking sufficient specific histological features to allow further classification. In this heterogeneous group of lesions, one has to exclude skin metastasis from deep organ adenocarcinoma, including salivary gland tumors metastasizing to the skin [18, 19, 20]. Further progress in the classification of these adnexal adenocarcinoma NOS may arise from molecular discoveries.

In conclusion, we report the first case of primary cutaneous adenocarcinoma with *PKRD3* rearrangement, suggesting that a cutaneous counterpart of the PAC/CASG entity may exist.

## Ethics Statement

This case report was conducted in accordance with institutional guidelines and the patient has given his consent.

## Conflicts of Interest

The authors declare no conflicts of interest.

## Data Availability

The data that support the findings of this study are available from the corresponding author upon reasonable request.
